# Exploring Greek midwives' knowledge, attitudes, and practices in perinatal smoking: A cross-sectional study

**DOI:** 10.18332/tpc/205916

**Published:** 2025-07-23

**Authors:** Taxiarchoula Delakovia, Paraskevi Katsaounou, Antigoni Sarantaki, Angeliki Bakou, Aikaterini Lykeridou, Athina Diamanti

**Affiliations:** 1 Department of Midwifery, School of Health and Care Sciences, University of West Attica, Athens, Greece; 2 Medical School, National and Kapodistrian University of Athens, Athens, Greece

**Keywords:** pregnancy, smoking cessation, midwives, fetal health, perinatal care, education

## Abstract

**INTRODUCTION:**

Smoking during pregnancy remains a significant global public health concern. Midwives, as frontline healthcare providers, play a crucial role in supporting smoking cessation among pregnant women. This study aimed to explore the factors associated with the implementation of smoking practices by midwives in Greece.

**METHODS:**

We conducted a cross-sectional survey between December 2022 and December 2023 among 150 midwives working in the 1st and 2nd Health Districts of Greece, as well as in private practices. Data were collected using a self-administered, anonymized questionnaire assessing demographics, knowledge, opinions, perceptions, and smoking cessation practices. Composite scores were calculated for knowledge, opinions, perceptions, and practices. Exploratory factor analysis was applied to examine the structure of midwives’ opinions and perceptions. Multivariate linear regression was used to identify independent predictors of smoking cessation practices.

**RESULTS:**

Among the respondents, 78% had not received formal education on smoking cessation, although 77.3% expressed a desire for training. Only 35.3% recognized that secondhand smoke affects newborns, and 32.7% felt confident in supporting pregnant women to quit smoking. Factor analysis identified two key dimensions: midwives’ contribution to smoking cessation during pregnancy, and information and help to stop smoking during pregnancy, explaining 27.7% and 16.9% of the variance, respectively. Multivariate regression revealed that both factors were independently associated with more frequent implementation of smoking cessation practices (p<0.05).

**CONCLUSIONS:**

The study underscores the pivotal role of midwives in smoking cessation during pregnancy and highlights the importance of perceptions, self-efficacy, and access to information in shaping their practices. Targeted education and institutional support are essential to strengthen midwives’ capacity to deliver effective smoking cessation interventions. These findings can inform policy and training programs aiming to improve maternal and neonatal outcomes by reducing tobacco use during pregnancy.

## INTRODUCTION

Smoking during pregnancy continues to pose a significant global public health challenge, presenting substantial risks to the health of both mothers and fetuses. Throughout the perinatal period, exposure to tobacco smoke stands out as the most significant preventable contributor to numerous adverse pregnancy outcomes^1,2^.

In Greece, smoking is notably prevalent among the population, with data from the 2020 Eurobarometer indicating a tobacco and related products usage rate of 42%^3^. Recent surveys conducted within Greece underscore the persistent nature of smoking during pregnancy as a serious public health issue^4,5^. One survey revealed that at the onset of pregnancy, 46.7% of expectant mothers identified themselves as smokers, with 17.5% continuing to smoke throughout their pregnancies^4^. Another recent study found that 41.4% of participating pregnant women reported smoking, while the overall prevalence of smoking at the conclusion of pregnancy stood at 19.7%^5^.

Smoking during pregnancy significantly impacts maternal and fetal health, affecting various stages of development and postnatal outcomes. Maternal smoking is associated with decreased fertility, increased risk of ectopic pregnancy, spontaneous abortion, and various placental pathologies, including altered blood flow and reduced placental function. Additionally, exposure to nicotine and carbon monoxide from cigarette smoke can result in lower birth weights, preterm births, and increased risks of stillbirth and sudden infant death syndrome (SIDS)^6^.

Efforts to reduce smoking during pregnancy are crucial for improving both immediate and long-term health outcomes for both mothers and their children. Given their constant interaction with pregnant women during both the prenatal and postpartum periods, midwives play a direct role in supporting smoking cessation effort^7^. Midwives play a pivotal role in supporting women through pregnancy and childbirth, advocating for healthy behaviors, and providing essential guidance on smoking cessation. As frontline healthcare professionals, their attitudes, knowledge, and perceptions regarding smoking in pregnancy profoundly influence the care provided to expectant mothers^8^. Moreover, with the evolution of smoking cessation practices, understanding midwives’ perspectives on newer strategies becomes imperative for improving maternal and neonatal outcomes^8^.

In recent years, the landscape of smoking cessation has witnessed the emergence of innovative strategies leveraging technology and behavioral science. From mobile health applications to virtual counseling platforms, these novel approaches offer promising avenues for enhancing engagement and adherence to smoking cessation interventions among pregnant women^9^. Midwives’ awareness of these advancements and their willingness to adopt them into practice are critical for addressing the evolving needs of expectant mothers and improving smoking cessation outcomes in pregnancy.

Primary prevention strategies focus on averting smoking initiation among women of childbearing age, thereby mitigating the risks associated with smoking during pregnancy. Secondary prevention involves early detection and intervention for pregnant women who smoke, aiming to minimize the adverse effects on maternal and fetal health. Tertiary prevention strategies focus on minimizing harm and optimizing outcomes for pregnant women who continue to smoke despite cessation support^10,11^. Midwives’ perceptions of smoking cessation approaches, such as nicotine replacement therapy, shape their recommendations and support strategies for women struggling to quit smoking during pregnancy^12^.

The aim of this study is to explore factors that are associated with implementation of smoking cessation practices by midwives.

## METHODS

### Study design

We conducted a cross-sectional survey among Greek midwives working in health centers and hospitals in the 1st and 2nd Health Districts or at private practices, from December 2022 to December 2023, using an online self-administered questionnaire. In a previous study, the authors evaluated the attitudes and knowledge of midwives about smoking cessation perinatally^13^. The sample for the collection of the data consisted of midwives (n=170) working in the aforementioned health services. The inclusion criteria were: 1) age >18 years, 2) agreement to participate in the study, 3) sufficient knowledge of the Greek language, and 4) practicing midwifery care. The exclusion criterion was a midwife’s involvement in professional practices linked to smoking cessation. A total of 150 midwives fully responded to the questionnaire (88.2%). The response rate was by tracking the number of invitations sent through direct emails and estimating the reach within closed midwife groups.

### Data collection

We created the self-administered anonymized questionnaire which we shared through online closed groups where midwives participate, and via personal e-mail to midwives. The questionnaire was designed to be specific to midwives, and the initial screening questions verified the respondents’ profession and practice settings. Participation in the survey was voluntary. At the outset of the questionnaire, a brief paragraph was provided to inform participants about the study’s aims and assure them of the confidentiality of their responses. All participants gave informed consent. Data were collected anonymously. The study protocol was approved by the Clinical Research and Ethics Committee of the 1st and 2nd Health Districts (protocol numbers 68197/15-11-2022 and 50502/23-11-2022, respectively) and by the Research Ethics Boards of the University of West Attica (protocol number 64102/05-07-2023).

### Questionnaire

The questionnaire was based on evidence-based sources, including peer-reviewed studies and national guidelines on tobacco control^7,14,15^. It underwent pilot testing with a small sample of midwives to assess clarity, relevance, and format. Feedback from the pilot test led to necessary revisions, ensuring the questionnaire’s reliability and validity for the main survey. We divided the questionnaire (Supplementary file) into 7 Sections: demographics of the study population; education about smoking and quitting smoking; smoking status of the participants; knowledge about smoking and smoking cessation in pregnant women; opinions about smoking and smoking cessation in pregnant women; practices for smoking cessation in pregnant women; and perceptions about smoking and smoking cessation in pregnant women.

### Outcome

In order to measure our primary outcome, we calculated a score related to the smoking cessation practices by midwives (using responses from Section 6, item 35 of the questionnaire), which evaluated the frequency of smoking cessation activities undertaken by midwives during prenatal care. Midwives indicated how often they performed each practice on a 5-point scale from 0 (‘Never’) to 4 (‘Very often’). The average of these items produced the final practices score, with higher values representing more frequent implementation of smoking cessation practices.

### Measures

We collected demographic data of the study population. These included: age, sex, nationality, family status (married, not married), education level (Master’s degree/Doctorate, Bachelor’s degree/School of Midwives), work experience in the field of health in years and work setting (Primary healthcare, Secondary healthcare, Tertiary healthcare, private practice).

We calculated a knowledge score, reflecting midwives’ factual understanding of smoking health harms during pregnancy. This score was calculated based on 14 items (Section 4, items 20 to 33), which assessed participants’ understanding of the health effects of smoking during pregnancy. Each correct answer was awarded one point. The total score was then standardized to range from 0 to 100, with higher scores indicating a higher level of smoking’s consequences during pregnancy.

An opinions score was also evaluated (Section 5, item 34 of the questionnaire), which asked midwives to rate their level of agreement with statements regarding the impact of smoking, including third hand smoke and partner influence. Responses were rated on a 5-point Likert scale ranging from 0 (‘Not at all’) to 4 (‘Extremely’). The final score was calculated as the average of all opinion-related items, with higher values reflecting recognition of the harmful health effects associated with smoking during pregnancy.

Finally, we also examined a perceptions score (Section 7, item 36), which explored midwives’ beliefs about their role and ability in smoking cessation support as well as perceptions about education adequacy and institutional infrastructure. Each statement was rated on a 5-point scale.

### Statistical analysis

Continuous variables were tested for normality using the Kolmogorov-Smirnov test. Descriptive statistics included means and standard deviations (SD) for normally distributed variables and medians with interquartile ranges for non-normally distributed variables. Categorical variables were summarized using absolute (n) and relative (%) frequencies. To assess internal consistency of the questionnaire and the composite scores used in the analysis, Cronbach’s alpha was calculated for each relevant section.

Exploratory factor analysis was applied to identify latent constructs underlying participants’ responses regarding their opinions, practices, and perceptions about smoking cessation. This technique was used to determine whether individual items are clustered into meaningful domains, thereby supporting the construct validity of the composite scores. We applied the Varimax rotation method and evaluated sample adequacy with the Kaiser-Meyer-Olkin (KMO) measure and Bartlett’s test of sphericity.

Multivariate linear regression analyses were conducted to identify independent predictors of our main outcome (practices of smoking cessation activities in pregnancy by midwives). Independent variables included demographic characteristics (age, gender, marital status, education level, work setting and work experience in the field of health), smoking status, prior education about smoking cessation and the scores related to knowledge about smoking and smoking cessation in pregnant women, opinions about smoking and smoking cessation in pregnant women, and perceptions about smoking and smoking cessation in pregnant women, as well as parameters identified by the factor analysis. The results of the multivariate linear regression analysis are presented as dependence coefficients (β), standard errors (SE), standard coefficients (b) and p-values for each independent variable. When the dependent variable did not meet the assumption of normality, it was log-transformed. We used SPSS version 26.0 for all statistical analyses. Two-sided significance tests were used, and statistical significance was set at p<0.05.

## RESULTS

### Questionnaire responses

Demographic characteristics of the study population, prior smoking cessation education, smoking status, knowledge, opinions on smoking cessation and factor analysis results are given in Supplementary file Tables 1–9. Notably, these results suggest that 78% of the respondents answered that they have not had any education about smoking and quitting smoking, while 77.3% stated that they would like to be educated at smoking cessation. At the same time, most midwives (55.3%) identified as never smokers, whereas 19.3% stated that they smoke daily, and 12% were occasional smokers. As far as opinions on the harmful effects of smoking during pregnancy are concerned, only 35.3% of midwives recognized that secondhand smoking affects the newborn. Furthermore, the perception of midwives regarding their role in smoking cessation varied, with some (32.7%) expressing that they feel very or extremely confident in their ability to support pregnant women in quitting smoking, while most felt not at all, slightly or moderately capable (77.3%).

Our factor analysis identified two distinct factors. The first, ‘Midwives’ contribution to smoking cessation during pregnancy’, comprised 6 items, explained 27.7% of the variability. The second, ‘Information and help to stop smoking during pregnancy’ consisted of nine questions and explained 16.9% of the variability.

Subsequently, taking into account the above two parameters of the factor analysis, we performed multivariate linear regression with the practices score as the dependent variable, participants’ demographic characteristics, smoking cessation education, smoking status, their knowledge of the effects on pregnancy, and their opinions and perceptions about it, as independent variables to identify the factors independently associated with smoking cessation practices during pregnancy. We found an independent relationship between smoking cessation practices and variables regarding perceptions of the importance of midwives’ contribution to smoking cessation during pregnancy (dependence coefficient β=0.052, standard error SE=0.009, standard coefficient b=0.434, p<0.001), as well as information and assistance on this topic (dependence coefficient β=0.028, standard error (SE)=0.016, standard coefficient b=0.133, p=0.047) (Table 1).

**Table 1:** Multivariate linear regression with the practices score as the dependent variable and participants’ demographic characteristics, smoking cessation education, smoking status, knowledge of the effects on pregnancy, and associated opinions and perceptions as independent variables, December 2022 – December 2023 (N=150)

*Variables*	*β*	*b*	*SE*	*p*
**Sociodemographic**				
Gender (females vs male)	0.005	0.043	0.008	0.906
Age	0.002	0.002	0.219	0.322
Married (yes vs no)	0.007	0.017	0.036	0.675
Education level (Master’s degree/Doctorate vs Bachelor’s degree/School of Midwives)	0.006	0.014	0.031	0.662
Work experience in the field of health (years)	0.000	0.002	0.017	0.937
**Work setting**				
Primary healthcare vs private practice	−0.005	0.022	−0.024	0.829
Secondary healthcare vs private practice	−0.023	0.025	−0.086	0.368
Tertiary healthcare vs private practice	0.024	0.024	0.097	0.311
**Smoking**				
Do you have some education about smoking and quitting smoking (yes vs no)	0.024	0.018	0.101	0.194
Smoker (yes vs no)	−0.023	0.016	−0.107	0.146
Knowledge score	0.001	0.001	0.005	0.950
Opinions of the participants about smoking during pregnancy	0.003	0.012	0.019	0.811
Midwives’ contribution to smoking cessation during pregnancy	0.052	0.009	0.434	**<0.001**
Information and help to quit smoking during pregnancy	0.028	0.016	0.133	**0.047**

β: dependence coefficient. b: standard coefficient. SE: standard error. The logarithm of the dependent variable has been used.

## DISCUSSION

This study aimed to explore factors that are associated with implementation of smoking cessation practices by midwives to shed light on various aspects of midwifery care related to smoking cessation interventions in Greece. This focus is especially relevant given the limited number of comprehensive training programs for healthcare professionals, including midwives, in smoking cessation interventions^8,14,16,17^.

Midwives play a key role in providing prenatal care, and their understanding of the risks of smoking during pregnancy directly influences the quality of care they provide to pregnant women. Studies have shown that midwives with higher levels of knowledge about the effects of smoking during pregnancy are more likely to engage in smoking cessation practices and provide effective counseling to pregnant women^18,19^. Therefore, efforts to enhance midwives’ education and training in smoking cessation interventions are essential to improve patient outcomes and reduce the prevalence of smoking during pregnancy.

The prevalence of smoking among midwives in this study was relatively low, with the majority being non-smokers. However, a notable proportion had previously smoked, indicating personal experience with tobacco use that could potentially inform their counseling approaches. It is encouraging to note that most midwives frequently inquire about smoking status and provide counseling to pregnant women^20^. These findings suggest a commitment to addressing smoking cessation within the scope of midwifery practice.

Previous research has demonstrated the positive impact of midwives’ engagement in smoking cessation practices on smoking behavior among pregnant women^21-23^. Research has shown that midwives’ advice and support can increase quit rates and reduce smoking relapse during pregnancy^24-26^. As a result, this study highlights the importance of including smoking cessation strategies into standard prenatal care.

The attitudes and perceptions of midwives play a crucial role in shaping their approach to smoking cessation interventions. Our results suggest that even though the majority of midwives recognized the harmful effects of smoking during pregnancy, there were some misconceptions and gaps in knowledge, particularly regarding thirdhand smoke exposure and the use of other tobacco products. These findings underscore the necessity of continuous education and training to reduce misconceptions and enhance midwives’ knowledge of tobacco-related risks resulting not only from active smoking but also from secondhand and thirdhand smoking^27^.

Furthermore, the perception of midwives regarding their role in smoking cessation varied, with some expressing confidence in their ability to support pregnant women in quitting smoking, while others felt less capable. This discrepancy underscores the importance of addressing self-efficacy and providing midwives with the necessary skills and resources to deliver effective smoking cessation interventions^28,29^. These findings are consistent with previous research indicating a direct relationship between midwives’ knowledge levels and their attitudes towards smoking cessation^30,31^.

The study also examined the factors influencing midwives’ implementation of smoking cessation practices during prenatal care and possible facilitators. The study identified perceptions of midwives’ contribution to smoking cessation and the availability of information and assistance on cessation as independent predictors of the implementation of cessation practices. Midwives who recognized the significance of their role in supporting smoking cessation efforts and had access to resources and guidance on cessation interventions, were more likely to engage in cessation practices.

Midwives often have limited time during prenatal appointments to address smoking cessation adequately. Midwives may encounter challenges in addressing smoking cessation due to competing priorities during prenatal visits, such as monitoring fetal health, discussing birth plans, or addressing other health concerns. However, building trusting relationships, providing non-judgmental support, offering tailored counseling, providing access to resources and referrals, and collaborating with interdisciplinary teams have all been mentioned as facilitators for providing smoking cessation advice during pregnancy^8,15,22,30,31^. The above are important as midwives knowledge on smoking cessation is directly associated with their attitudes towards smoking cessation^32,33^.

### Limitations

It is important to acknowledge several limitations of the study. Firstly, the study was conducted only in Attica, posing geographical restrictions on the final conclusions. Moreover, this is a cross-sectional study. Cross-sectional studies capture data at a single point in time, which limits the ability to establish temporal relationships or causality. In addition, the reliance on self-reported data may introduce biases such as social desirability bias and recall errors. Moreover, the relatively small sample size restricts the generalizability of the findings to the wider population of midwives. Thus, future research should endeavor to validate these results using larger and more diverse samples to ensure the reliability and applicability of the conclusions.

## CONCLUSIONS

This study identified key factors associated with the capacity to deliver effective smoking cessation interventions by midwives’ in clinical care. Despite the overall recognition of smoking’s harmful effects, important gaps in knowledge and self-efficacy remain among midwives in Greece, underlining the need for enhanced training and institutional support. Notably, perceptions about their role and access to relevant information were independently associated with more frequent engagement in smoking cessation activities. Future research should aim to validate these findings in broader populations and explore the long-term impact of targeted interventions in midwifery education and practice.

## Supplementary Material



## Data Availability

The data supporting this research are available from the authors on reasonable request.
