# Supplementary material for: Exploring Greek midwives' knowledge, attitudes, and practices in perinatal smoking: A cross-sectional study
Source: Tob Prev Cessat. 2025 Jul 23;11:10.18332/tpc/205916. doi: 10.18332/tpc/205916 (PMC12284828; doi:10.18332/tpc/205916)
Supplement: Supplementary file 1 [file TPC-11-33-s1.pdf]

QUESTIONNAIRE:

Research on attitudes, knowledge, and perceptions of midwives for smoking.

SECTION 1

DEMOGRAPHICS

1. Age (years):

[ ]

2. Sex:

- Male
- Female
- Other

3. Nationality:

[ ]

4. Family status:

- Single
- Married
- Cohabilitation agreement
- Divorced
- Widowed

5. Level of education:

- Schools of midwives
- Bachelor’s degree
- Postgraduate
- Doctorate
- Postdoctorate

6. Work experience in the field of health in years:

7. Work setting

- Primary health care
- Secondary health care
- Tertiary health care
- Private practice

SECTION 2

EDUCATION ABOUT SMOKING AND QUITTING SMOKING

8. Do you have some education about smoking and quitting smoking?

- Yes
- No

9. Would you like to be educated at smoking cessation?

- Yes
- No

10. What do you consider the best type of this particular education? ***(You can choose more than one answer)***

- As separate and specific courses of smoking cessation in undergraduate level.
- As part of existing courses in undergraduate level.
- As separate and specific courses of smoking cessation in postgraduate level.
- As special seminar (e.g. for of lifelong learning)
- Other

11. What would you like to be the purpose of the training in smoking cessation? ***(You can choose more than one answer)***

- Understanding of risks such for the pregnant as well as for the fetus and its later life because of its exposure to the smoke of a cigarette
- Understanding the mechanism of nicotine addiction and education at techniques for smoking cessation.
- Understanding and training in behavior therapy, motivation/ interview and at short counseling.
- Other

12. Answer the following questions (Not *at all, Slightly, Moderately, Very, Extremely)*

	Not at all	Slig htly	Moderately	Very	Extremely
**To what extent do you consider your education in smoking cessation techniques useful in undergraduate level?**	☐	☐	☐	☐	☐
**To what extent do you consider your education in smoking cessation techniques useful in postgraduate level?**	☐	☐	☐	☐	☐
**To what extent do you think that receiving a training in smoking cessation techniques would help you to your daily clinic exercise to be able to help pregnant and lying-in women who smoke to quit smoking?**	☐	☐	☐	☐	☐

SECTION 3

SMOKING STATUS

13. Which is your smoking status?

- Never smoker
- Ex-smoker
- Smoking daily
- Occasional smoking

14. Determine the number of cigarettes you consume daily ***(answer only if you are smoker)*** **……………………**

15. How many years have you been smoking? ***(answer only if you aresmoker)***

………………

16. At what age did you start smoking? ***(answer only if you are a smoker)***
………………

17. How soon after you wake up do you smoke your first cigarette ***(answer only if you are smoker)***

- After 60 minutes
- Within 31-60 minutes
- Within in 6-30 minutes
- Within the first five minutes

18. Do you use ***(answer only if you are a smoker, you can choose more than one answer)***

- Conventional cigarette
- Heated tobacco
- Electronic cigarette
- Other

19. Have you tried to quit smoking in the past? ***(answer only if you are smoker)***

- Yes
- No

SECTION 4

KNOWLEDGE

20. For which of the following situations do you consider smoking in pregnancy is a risk factor? ***(You can choose more than one answer)***

- Placenta detachment
- Premature rupture of membranes
- Premature childbirth
- Spontaneous abortions
- Asthma in childhood
- Congenital anomalies such as cleft lip and lycostoma
- Neurodevelopmental problems at newborns
- Syndrome of suddenly death
- Development of cancer in childhood
- Obesity in childhood

21. Symptoms may occur when quitting smoking such as headache, dizziness, weakness, anxiety and difficulty in concentration.

- Yes
- No

22. Smoking during pregnancy causes damage to the fetus independently of number of cigarettes.

- Yes
- No

23. Nicotine is equally addictive as heroin, as a factor in changing disposition and behavior.

- Yes
- No

24. What is the safe limit for cigarette consumption in pregnancy?

- 0 cigarettes
- 1-3 cigarettes
- 3-5 cigarettes
- Other

25. The use of new tobacco products (ENDS, e-cigarette, IQOS, etc.) is allowed during pregnancy.

- Yes
- No

26. Nicotine and carbon monoxide penetrate the placental barrier and are detected in the blood of the fetus at higher levels compared to the pregnant.

- Yes
- No

27. When quitting smoking during pregnancy, is it allowed to use nicotine replacement products?

- Yes
- No

28. Do know the term "motivator interview"?

- Yes
- No

29. Do you know models "5As" and "5Rs" as an intervention for smoking cessation?

- Yes
- No

30. Do you know what is the IQOS and the ENDS products?

- Yes
- No

31. Would you recommend the use others of tobacco products (e.g. electronic cigarette, heated tobacco) instead of conventional cigarette during pregnancy?

- Yes
- No

32. If you answered yes to the previous question, which would you recommend? and why? ……………………………................................... ....
…………………………………………………………………………………………………..

33. Do you know the smoking cessation services where you can refer the pregnant smokers?

- Yes
- No

SECTION 5

OPINIONS

34. Answer the following questions (Not *at all, Slightly, Moderately, Very, Extremely)*

	Not at all	Slightly	Moderately	Very	Extremely
**To what extent do you consider that the thirdhand exhibition to smoke (smoke that stays in clothes, objects etc.) affects the pregnant and the fetus?**	☐	☐	☐	☐	☐
**To what extent do you consider that the thirdhand exhibition to smoke (smoke that stays in clothes, objects etc.) affects the newborn and the child?**	☐	☐	☐	☐	☐
**To what extent do you consider that the smoking status of the pregnant's companion contributes to the pregnant's failure to quit smoking?**	☐	☐	☐	☐	☐
**To what extent do you consider that the attempt of the pregnant's companion to quit smoking affects the pregnant's success to quit smoking?**	☐	☐	☐	☐	☐

**To what extent do you consider that the milk of a pregnant smoker is affected by tobacco smoke?**	☐	☐	☐	☐	☐
**To what extent do you consider that smoking is harmful during breastfeeding so that it is preferable to not breastfeed the baby?**	☐	☐	☐	☐	☐

____

SECTION 6

PRACTICES

35. In the context of prenatal meetings how often

	Neve r	Rarely	A few times	Often	Very often
**Do you ask pregnant women if they smoke?**	☐	☐	☐	☐	☐
**Do you specify the smoking status of the pregnant (e.g. if they are active smokers, if they quit smoking due to pregnancy, if they quit the last 2 weeks, if they were smokers in the past, how many cigarettes do they smoke?)**	☐	☐	☐	☐	☐
**Do you ask the pregnant smokers how much they smoke?**	☐	☐	☐	☐	☐

**When taking obstetric history beyond of questions about with the conventional cigarette use do you ask the pregnant and lactating women if they use other tobacco products such as electronic cigarette, heated tobacco etc.?**	☐	☐	☐	☐	☐
**Do you ask the pregnant women if there are smokers in their family?**	☐	☐	☐	☐	☐
**Are you involved in efforts to quit smoking of pregnant women and smokers in her environment?**	☐	☐	☐	☐	☐
**Do you explain the dangers of smoking?**	☐	☐	☐	☐	☐
**Do you advise the pregnant smokers to quit smoking?**	☐	☐	☐	☐	☐
**Do you ask the pregnant smokers if they feel ready to quit smoking and set a date of quitting?**	☐	☐	☐	☐	☐
**Do you recommend to pregnant smokers that they start direct efforts for smoking cessation?**	☐	☐	☐	☐	☐

**Do you recommend to pregnant smokers to reduce smoking?**					
**Do you refer the pregnant smokers to smoking cessation services?**					

SECTION 7

PERCEPTIONS

36. Answer the following questions (Not at all, Slightly, Moderately, Very, Extremely)

	Not at all	A little bit	Enough	Very	Despite very
**Are pregnant women informed about the risks of firsthand, secondhand and thirdhand exposure to tobacco smoke?**					
**Could the counseling for smoking cessation affect my relationship with the pregnant/lying-in woman?**					
**Can smoking help the pregnant woman to manage her stress during pregnancy?**					

Do you agree that pregnant women__

**with mental illnesses are preferable to not quit smoking despite manifest withdrawal symptoms due to her physical dependence they have on the smoke of cigarettes?**					
**Midwives play an important role in informing the pregnant smokers about smoking cessation.**					
**Midwives play an important role in informing the lying-in women smokers about smoking cessation.**					
**Smoking cessation is a part of obstetrics care.**					
**Midwives don't have enough education about smoking cessation.**					
**The education of of midwives about smoking cessation should be included in undergraduate level.**					
**If there were smoking cessation settings at maternity hospitals and at obstetrics clinics it would be more easy to the recommend them**					

**to pregnant smokers**.					
**If there were smoking cessation settings at maternity hospitals and at obstetrics clinics the pregnant women would visit and trust them more easily.**	☐	☐	☐	☐	☐
**How important do you think the partner's involvement in smoking cessation?**	☐	☐	☐	☐	☐
**How capable do you feel to support the pregnant/lying-in women in smoking cessation?**	☐	☐	☐	☐	☐
**How confident do you feel to speak up at pregnant / lying-in women for the risks of smoking?**	☐	☐	☐	☐	☐
**How confident you feel to recommend to pregnant/lactating women to quit smoking?**	☐	☐	☐	☐	☐

Supplementary file 2

Table 1. Demographic characteristics of the study population (n=150, data collected December 2022- December 2023)

Variable		N	%
**Gender**	Male	4	2.7
	Female	146	97.3
**Age (years), Mean value (standard deviation), Median (interquartile range)**		39.9 (10.3)	40 (30 – 47)
**Nationality**	Greek	148	98.7
	Albanian	2	1.3
**Family status**	Single	51	34
	Married	92	61.3
	Cohabilitation agreement	1	0.7
	Divorced	6	4
	Widowed	0	0
**Level of education**	School of midwives	5	3.4
	Bachelor's degree	81	54.4
	Master's degree	55	36.9
	Doctorate	8	5.4
	Postdoctorate	0	0
**Work experience in the field of health in years, Mean value (standard deviation), Median (interquartile range)**		16 (9.7)	17 (7 – 23)
**Work setting**	Primary health care	71	47.3
	Secondary health care	25	16.7
	Tertiary health care	30	20
	Private practice	24	16

Table 2. Education of the participants in smoking cessation. (n=150, data collected December 2022- December 2023)

Question		N	%
**Do you have some education about smoking and quitting smoking?**	No	117	78
	Yes	33	22
**Would you like to be educated at smoking cessation?**	No	34	22.7
	Yes	116	77.3
**What do you consider the best type of this particular education?** ***(You can choose more than one answer)***			
As separate and specific courses of smoking cessation in undergraduate level.		36	24
As part of existing courses in undergraduate level.		35	23.3
As separate and specific courses of smoking cessation in postgraduate level.		15	10
As special seminar (e.g. for of life learning)		115	76.7
Other		3	2
**What would you like to be the purpose of the training in smoking cessation?** ***(You can choose more than one answer)***			
Understanding of risks such for the pregnant as well as for the fetus and its later life because of its exposure to the smoke of a cigarette		105	70
Understanding the mechanism of nicotine addiction and education at techniques for smoking cessation.		98	65.3
Understanding and training in behavior therapy, in motivation interview and at short counseling.		77	51.3
Other		2	1.3
**To what extent do you consider your education in smoking cessation techniques in undergraduate level?**	Not at all	6	4
	Slightly	15	10
	Moderately	57	38
	Very	36	24

	Extremely	36	24
**To what extent do you consider your education in smoking cessation techniques useful in postgraduate level?**	Not at all	7	4.7
	Slightly	18	12
	Moderately	57	38
	Very	35	23.3
	Extremely	33	22
**To what extent do you think that receiving a training in smoking cessation techniques would help you to your daily clinic exercise to be able to help pregnant and lying-in women who smoke to quit smoking?**	Not at all	1	0.7
	Slightly	9	6
	Moderately	37	24.7
	Very	35	23.3
	Extremely	68	45.3

Table 3. Smoking status of the participants. (n=150, data collected December 2022- December 2023)

**Question**		**N**	**%**
**Which is your smoking status?**	Never smoker	83	55.3
	Ex-smoker	20	13.3
	Smoking daily	29	19.3
	Occasional smoking	18	12
**Determine the number of cigarettes you consume daily. ***		10.2 (9)	9 (3 – 17)
**How many years have you been smoking? ***		18 (10)	19 (10 – 25)
**At what age did you start smoking? ***		21(6)	20 (18 – 22)
**How soon after you wake up do you smoke your first cigarette? ***	After 60 minutes	18	38.3
	Within 31-60 minutes	7	14,9
	Within 6-30 minutes	12	25.5

	Within the first five minutes	2	4.2
**Do you use: * *(You can choose more than one answer)***			
Conventional cigarette		27	57.4
Heated tobacco		16	34
Electronic cigarette		5	10.6
**Have you tried to quit smoking in the past? ***	No	17	36.2
	Yes	27	57.4

*Calculated only for smokers

Table 4. Knowledge about smoking and smoking cessation in pregnancy(n=150, data collected December 2022- December 2023)

Question		N	%
**For which of the following situations do you consider smoking in pregnancy is a risk factor? *(You can choose more than one answer)***			
1. Placenta detachment		98	65.3
2. Premature rupture of membranes		66	44
3. Premature childbirth		130	86.7
4. Spontaneous abortions		90	60
5. Asthma in childhood		95	63.3
6. Congenital anomalies such as cleft lip and lycostoma		32	21.3
7. Neurodevelopmental problems at newborns		89	59.3
8. Syndrome of suddenly death		87	58
9. Development of cancer in childhood		37	24.7
10. Obesity in childhood		17	11.3
**Symptoms may occur when quitting smoking such as headache, dizziness, weakness, anxiety and difficulty in concentration.**	No	5	3.3
	Yes	145	96.7

**Smoking during pregnancy causes damage to the fetus independently of number of cigarettes.**	No	23	15.3
	Yes	127	84.7
**Nicotine is equally addictive as heroin, as a factor in changing disposition and behavior.**	No	18	12
	Yes	132	88
**What is the safe limit for cigarette consumption in pregnancy?**	0 cigarettes	96	64
	1-3 cigarettes	48	32
	3-5 cigarettes	5	3,3
	Other	1	0.7
**Is the use of new tobacco products (ENDS, e-cigarette, IQOS, etc.) allowed during pregnancy?**	No	136	90.7
	Yes	14	9.3
**Nicotine and carbon monoxide penetrate the placental barrier and are detected in the blood of the fetus at higher levels compared to the pregnant.**	No	33	22
	Yes	117	78
**When quitting smoking during pregnancy, is it allowed to use nicotine replacement products?**	No	108	72
	Yes	42	28
**Do know the term "motivational interviewing"?**	No	114	76
	Yes	36	24
**Do you know models "5As" and "5Rs" as an intervention for smoking cessation?**	No	136	90.7
	Yes	14	9.3

**Do you know what IQOS, and the ENDS products is?**	No	33	22
	Yes	117	78
**Would you recommend the use others of tobacco products (e.g. electronic cigarette, heated tobacco) instead of conventional cigarette during pregnancy?**	No	144	96
	Yes	6	4
**If you answered yes to the previous question, which would you recommend? and why?**	E-cigarette	2	1.4
	IQOS due to reduced amount of nicotine	1	0.7
	IQOS as I also use it	1	0.7
	IQOS	1	0.7
**Do you know the smoking cessation services where you can refer the pregnant smokers?**	No	103	68.7
	Yes	47	31.3

*ENDS: electronic nicotine delivery systems

Table 5. Opinions of the participants regarding smoking during pregnancy. (n=150, data collected December 2022- December 2023)

Question		N	%
**To what extent do you consider that the thirdhand exhibition to smoke (smoke that stays in clothes, objects etc.) affects the pregnant and the fetus?**	Not at all	10	6.7
	Slightly	30	20
	Moderately	57	38
	Very	29	19.3
	Extremely	24	16
**To what extent do you consider that the thirdhand exhibition to smoke (smoke that stays in clothes, objects etc.) affects the newborn and the child?**	Not at all	7	4.7
	Slightly	17	11.3
	Moderately	51	34
	Very	33	22
	Extremely	42	28
**To what extent do you consider that the smoking status of the pregnant's companion contributes to the pregnant's failure to quit smoking?**	Not at all	3	2
	Slightly	5	3.3
	Moderately	32	21.3
	Very	47	31.3
	Extremely	63	42
**To what extent do you consider that the attempt of the pregnant's companion to quit smoking affects the pregnant's success to quit smoking?**	Not at all	0	0
	Slightly	4	2.7
	Moderately	31	20,7
	Very	41	27.3
	Extremely	74	49.3
**To what extent do you consider that the milk of a pregnant smoker is affected by tobacco smoke?**	Not at all	2	1.3
	Slightly	13	8.7
	Moderately	35	23.3
	Very	38	25.3
	Extremely	62	41.3
**To what extent do you consider that smoking is harmful during breastfeeding so that it is preferable to not breastfeed the baby?**	Not at all	26	17.3
	Slightly	35	23.3
	Moderately	39	26
	Very	19	12.7
	Extremely	31	20.7

Table 6. Exploratory factor analysis with Varimax rotation for opinions of the participants regarding smoking during pregnancy. (n=150, data collected December 2022- December 2023)

Question	Opinions of the participants regarding smoking during pregnancy
To what extent do you consider that thethirdhand exhibition to smoke (smoke that stays in clothes, objects etc.) affects the pregnant and the fetus	0.822
To what extent do you consider that the thirdhand exhibition to smoke (smoke that stays in clothes, objects etc.) affects the newborn and the child	0.789
To what extent do you consider that the smoking status of the pregnant's companion contributes to the pregnant's failure to quit smoking?	0.724
To what extent do you consider that the attempt of the pregnant's companion to quit smoking affects the pregnant's success to quit smoking?	0.701
To what extent do you consider that the milk of a pregnant smoker is affected by tobacco smoke?	0.440
To what extent do you consider that smoking is harmful during breastfeeding so that it is preferable to not breastfeed the baby?	0.405

Table 7. Practices of the participants regarding smoking cessation during pregnancy. (n=150, data collected December 2022- December 2023)

Question		N	%	% Often-Very often
**Do you ask pregnant women if they smoke?**	Never	2	1.3	84
	Rarely	3	2	
	A few times	16	10.7	
	Often	31	20.7	
	Very often	98	65.3	
**Do you specify the smoking status of the pregnant (e.g. if they are active smokers, if they quit smoking due to pregnancy, if they quit the last 2 weeks, if they were smokers in the past, how many cigarettes do they smoke?)**	Never	2	1.3	70
	Rarely	8	5.3	
	A few times	35	23.3	
	Often	41	27.3	
	Very often	64	42.7	
**Do you ask the pregnant smokers how much they smoke?**	Never	3	2	87.4
	Rarely	2	1.3	
	A few times	14	9.3	
	Often	34	22.7	
	Very often	97	64.7	
**When taking obstetric history beyond of questions about with the conventional cigarette**	Never	9	6	70
	Rarely	14	9.3	
	A few times	22	14.7	
	Often	32	21.3	

**use, do you ask the pregnant and lactating women if they use other tobacco products such as electronic cigarette, heated tobacco etc.?**	Very often	73	48.7	
**Do you ask the pregnant women if there are smokers in their family?**	Never	22	14.7	49.3
	Rarely	25	16.7	
	A few times	29	19.3	
	Often	26	17.3	
	Very often	48	32	
**Are you involved in efforts to quit smoking of pregnant women and smokers in her environment?**	Never	13	8.7	53
	Rarely	21	14	
	A few times	35	23.3	
	Often	43	28.7	
	Very often	38	25.3	
**Do you explain the dangers of smoking?**	Never	3	2	82
	Rarely	3	2	
	A few times	21	14	
	Often	34	22.7	
	Very often	89	59.3	
**Do you advise the pregnant smokers to quit smoking?**	Never	1	0.7	92
	Rarely	3	2	
	A few times	8	5.3	
	Often	39	26	
	Very often	99	66	
**Do you ask the pregnant smokers if they feel ready toquit smoking and set a date of quitting?**	Never	12	8	57.4
	Rarely	26	17.3	
	A few times	26	17.3	
	Often	34	22.7	
	Very often	52	34.7	

**Do you recommend to pregnant smokers that they start direct efforts for smoking cessation?**	Never	4	2.7	82.6
	Rarely	4	2.7	
	A few times	18	12	
	Often	47	31.3	
	Very often	77	51.3	
**Do you recommend to pregnant smokers to reduce smoking?**	Never	3	2	85.3
	Rarely	4	2.7	
	A few times	15	10	
	Often	29	19.3	
	Very often	99	66	
**Do you refer the pregnant smokers to smoking cessation services?**	Never	24	16.1	39.6
	Rarely	34	22.8	
	A few times	32	21.5	
	Often	23	15.4	
	Very often	36	24.2	

Table 8. Perceptions of the participants regarding smoking cessation during pregnancy. (n=150, data collected December 2022- December 2023)

Question		N	%	% Very-Extremely
**Are pregnant women informed about the risks of firsthand, secondhand and thirdhand exposure to tobacco smoke?**	Not at all	20	13.3	8.6
	Slightly	73	48.7	
	Moderately	44	29.3	
	Very	11	7.3	
	Extremely	2	1.3	
**Could the counseling for smoking cessation affect my relationship with the pregnant/lying-in woman?**	Not at all	25	16.7	14
	Slightly	51	34	
	Moderately	53	35.3	
	Very	14	9.3	
	Extremely	7	4.7	
**Can smoking help the pregnant woman to manage her stress during pregnancy?**	Not at all	37	24.7	11.3
	Slightly	56	37.3	
	Moderately	40	26.7	
	Very	14	9.3	
	Extremely	3	2	
**Do you agree that pregnant women with mental illnesses are preferable to not quit smoking despite manifest withdrawal symptoms due to her physical dependence they have on the smoke of cigarettes?**	Not at all	22	14.7	20
	Slightly	43	28.7	
	Moderately	55	36.7	
	Very	17	11.3	
	Extremely	13	8.7	
**Midwives play an important role in informing the pregnant smokers about smoking cessation.**	Not at all	1	0.7	71.3
	Slightly	3	2	
	Moderately	39	26	
	Very	32	21.3	
	Extremely	75	50	
**Midwives play an important role in informing the lying-in childbearing women**	Not at all	1	0.7	72.7
	Slightly	9	6	

**smokers about smoking cessation.**	Moderately	30	20	
	Very	31	20.7	
	Extremely	79	52.7	
**Smoking cessation is a part of midwifery care.**	Not at all	5	3.3	64
	Slightly	15	10	
	Moderately	34	22.7	
	Very	37	24.7	
	Extremely	59	39.3	
**Midwives don't have enough education about smoking cessation.**	Not at all	22	14.7	42.6
	Slightly	31	20.7	
	Moderately	33	22	
	Very	29	19.3	
	Extremely	35	23.3	
**The education of of midwives about smoking cessation should be included in undergraduate level.**	Not at all	3	2	68
	Slightly	11	7.3	
	Moderately	34	22.7	
	Very	34	22.7	
	Extremely	68	45.3	
**If there were smoking cessation settings at maternity hospitals and at maternity clinics it would be easier to the recommend them to pregnant smokers.**	Not at all	0	0	88
	Slightly	0	0	
	Moderately	18	12	
	Very	34	22.7	
	Extremely	98	65.3	
**If there were smoking cessation settings at maternity hospitals and at maternity clinics the pregnant women would visit and trust them more easily.**	Not at all	0	0	77.4
	Slightly	4	2.7	
	Moderately	30	20	
	Very	52	34.7	
	Extremely	64	42.7	
**How important do you think the partner's involvement insmoking cessation?**	Not at all	0	0	90.7
	Slightly	0	0	
	Moderately	14	9.3	
	Very	37	24.7	

	Extremely	99	66	
**How capable do you feel to support the pregnant/childbearing women in smoking cessation?**	Not at all	9	6	32.7
	Slightly	43	28.7	
	Moderately	49	32.7	
	Very	22	14.7	
	Extremely	27	18	
**How confident do you feel to speak up at pregnant / childbearing women for the risks of smoking?**	Not at all	3	2	50
	Slightly	20	13.3	
	Moderately	52	34.7	
	Very	38	25.3	
	Extremely	37	24.7	
**How confident you feel to recommend to pregnant/lactating women to quit smoking?**	Not at all	3	2	53.3
	Slightly	18	12	
	Moderately	49	32.7	
	Very	38	25.3	
	Extremely	42	28	

Table 9. Multivariate linear regression analysis with knowledge score as the dependent variable and participants' demographic characteristics, smoking cessation education, and smoking status as independent variables. (n=150, data collected December 2022- December 2023)

**Variable**	**β+**	**SE++**	**b***	**P**
**Age**	0.001	0.003	-0.018	0.940
**Gender (Females vs. males)**	0.046	0.070	0.051	0.518
**Work experience in the field of health in years**	-0.002	0.004	-0.108	0.653
**Do you have some education about smoking and quitting smoking (Yes vs. No)**	0.114	0.028	0.331	**<0.001**
**Married (Yes vs. No)**	0.048	0.028	0.161	0.089
**Level of Education (Master's degree/Doctorate vs. Bachelor's degree/School of midwives)**	0.018	0.023	0.060	0.449
**Work setting**				
**Secondary health care vs. Private practice**	-0.009	0.041	-0.024	0.818
**Tertiary health care vs. Private practice**	0.015	0.038	0.042	0.691
**Primary health care vs. Private practice**	-0.042	0.035	-0.147	0.234
**Smoking (Yes vs. No)**	-0.041	0.024	-0.133	0.093

+dependence coefficient ++standard error *standard coefficient; *note: The logarithm of the dependent variable has been used.*

© 2025 Delakovia T. et al.
